# Sodium Hypochlorite as an Adjunct to Nonsurgical Treatment of Periodontitis: A Systematic Review

**DOI:** 10.3290/j.ohpd.a45405

**Published:** 2020-10-13

**Authors:** Egle Ramanauskaite, Vita Machiulskiene, Meizi Eliezer, Anton Sculean

**Affiliations:** a PhD Student, Clinic of Dental and Oral Pathology, Lithuanian University of Health Sciences, Kaunas, Lithuania. Literature search, wrote the manuscript.; b Professor, Clinic of Dental and Oral Pathology, Lithuanian University of Health Sciences, Kaunas, Lithuania. Literature search, proofread the manuscript.; c Periodontist, Department of Periodontology, University of Bern, Switzerland; PerioHome, Private Clinic, Herzliya, Israel. Proofread the manuscript.; d Professor and Chair, Department of Periodontology, University of Bern, Switzerland. Idea, advisor, proofread the manuscript.

**Keywords:** periodontitis, review, therapy, treatment

## Abstract

**Purpose:**

To evaluate effects of the adjunctive subgingival application of sodium hypochlorite on clinical outcome following nonsurgical periodontal treatment.

**Materials and Methods:**

A search protocol was developed to answer the following focused question: ‘in patients with periodontitis, does adjunctive subgingival application of sodium hypochlorite have additional clinical benefits compared to subgingival debridement alone?’ Randomised controlled clinical trials (RCTs) published up to January 30, 2020, with at least 6 months of follow-up, in which sodium hypochlorite was used as an adjunct in nonsurgical periodontitis treatment were included. The search was limited to the English language.

**Results:**

Out of 355 studies retrieved, the search resulted in two publications that fulfilled the inclusion criteria. The adjunctive application of sodium hypochlorite did not provide additional beneficial effect in terms of changes in the evaluated clinical outcomes (i.e. probing depth values [PDs], clinical attachment level gain [CAL] and bleeding on probing [BOP]) when compared to mechanical instrumentation alone over the 12-month investigation period (p > 0.05).

**Conclusion:**

The available data have failed to show any additional clinical benefit following the use of sodium hypochlorite in conjunction with nonsurgical periodontal therapy.

Periodontitis is a chronic, multifactorial inflammatory disease associated with dysbiotic plaque biofilms, that results in progressive loss of attachment and bone.^30^ The number of people affected by periodontitis has grown substantially, increasing the total burden of disease globally.^15^ Periodontitis is a major public health problem because it may lead to tooth loss and disability, negatively affect chewing function and aesthetics, and impair quality of life.^28^

The incidence of incipient periodontal destruction increases with age, with periodontal pocketing as a principal state of destruction.^12^ Periodontal pockets contain biofilms of great complexity and are lined by inflamed epithelium.^26,39^

The goals of periodontal therapy include arresting disease progression, establishing healthy, stable, maintainable periodontal conditions and, if possible, regenerating the lost tissues.^8^ Furthermore, periodontal treatment should establish favourable surfaces on the periodontally involved teeth for new connective tissue attachment and repopulation of cells originating from the periodontal ligament.^27^

Cause-related therapy includes a thorough removal of supra- and subgingival biofilms. In patients with periodontitis, subgingival debridement is an effective treatment in reducing probing pocket depth and improving the clinical attachment level; however, it has limitations.^38^ It has been found that up to 30% of treated roots harbour residual plaque or calculus, resulting in microbial recolonisation, thus limiting the effects of therapy.^4,10,21,29,31,33^

A recent systematic review showed that antiseptics may be beneficial in treating patients with periodontitis.^32^ Antiseptics are chemical agents that can destroy microorganisms on live tissues. They are characterised by having a more extensive spectrum of activity compared to systemic or local antibiotics. Furthermore, the possibility of resistance formation is reduced by having multiple intracellular targets.^34,37^

Among the supragingivally used antiseptics, chlorhexidine remains a ‘gold standard’ in plaque control. It exhibits bactericidal activity and inhibits plaque regrowth; however, the adverse effects include discolouration of teeth, tongue, and restorations, increased formation of calculus and altered taste sensation.^14,20^

An alternative antiseptic material, sodium hypochlorite (NaOCl), has many properties of an ideal antimicrobial agent, including broad antimicrobial activity, rapid bactericidal action, relative nontoxicity, no colour, no staining and ease of access. Hypochlorite is lethal for most bacteria, fungi, and viruses.^37^

Sodium hypochlorite belongs to the chlorine-releasing agents group. It is a highly active oxidising agent, destroying cellular activity of proteins. In water, NaOCl ionises to produce Na^+^ and the hypochlorite ion OCl^-^, which establishes an equilibrium with hypochlorous acid (HOCl^-^), a key microbicidal agent.^23^ Hypochlorous acid causes irreversible enzymatic inactivation in bacteria and oxidises and disrupts the cell membrane, cell wall, and various macromolecules of microorganisms.^3^ Sodium hypochlorite is produced by activated human neutrophils and macrophages and plays an important role in the innate immune system.^11^

Until now, there has been no review addressing the effectiveness of sodium hypochlorite in nonsurgical treatment of periodontitis. Therefore, the aim of the current article is to investigate the current knowledge of the clinical effects of adjunctive subgingival use of sodium hypochlorite in the treatment of periodontitis.

## Materials and Methods

The reporting of this systematic analysis adhered to the Preferred Reporting Items for Systematic Review and Meta-Analyses (PRISMA) statement.^25^

### Protocol and Registration

The review was registered in PROSPERO, an international prospective register of systematic reviews, under number CRD42017063950. The methods of analysis and inclusion criteria were specified in advance and documented in a protocol, accessible through the following link: https://www.crd.york.ac.uk/PROSPERO/display_record.php?ID=CRD42017063950

### Focus Question

The following focus question was developed according to population, intervention, comparison, outcome and study design (PICOS): ‘in patients with periodontitis does adjunctive subgingival application of sodium hypochlorite have additional clinical benefits compared with subgingival debridement alone?’

- (P)opulation: Systemically healthy patients, older than 18 years, diagnosed with untreated periodontitis, or patients with recurrent periodontits, enrolled in regular periodontal maintenance programs;
- (I)ntervention (test): SRP plus adjunctive subgingival application of sodium hypochlorite;
- (C)omparison (control): SRP alone or plus a placebo;
- (O)utcome: The primary outcome variable was change in pocket-probing depths (PD); secondary outcome variables included changes in clinical attachment level (CAL) and/or bleeding on probing (BOP) and/or incidence of adverse events;
- (S)tudy design and duration: Randomised controlled clinical trials (RCTs) with parallel or split-mouth designs with a minimum duration of 6 months.

### Search Strategy

MEDLINE (Ovid), EMBASE, and the Cochrane Central Register of Controlled Trials (CENTRAL) databases were searched for relevant articles published up to 30 January, 2020. The search was limited to human studies and the English language.

In addition, a hand search was performed including reference lists of all full-text articles and the following scientific journals: International Journal of Periodontics and Restorative Dentistry, Journal of Clinical Periodontology, Journal of Periodontology, and Journal of Periodontal Research. The search terms used are given below.

- Population: “chronic periodontitis” [MeSH term] OR “periodontal disease” [MeSH term] OR “periodontitis” [MeSH term]
- Intervention: “sodium hypochlorite” [MeSH term] OR “hypochlorite” [MeSH term] OR “treatment” [MeSH term] OR “periodontal therapy” [MeSH term] OR “scaling and root planning” [MeSH term] OR “subgingival irrigation” [MeSH term]
- Population AND Intervention

### Selection of Studies

Titles and abstracts were independently screened by two authors (ER and VM) based on the inclusion criteria. Further, full texts were read to confirm each study’s eligibility based on the inclusion and exclusion criteria stated below. Any disagreements were solved through discussion until a consensus was reached and by consulting an experienced third reviewer (AS). The agreement level between the reviewers regarding study inclusion was calculated using κ (kappa) statistics.

### Inclusion and Exclusion Criteria

During the first stage of study selection, the titles and abstracts were screened and evaluated according to the following inclusion criteria: randomised controlled clinical trials where periodontitis patients received nonsurgical treatment; parallel and split-mouth design studies including systemically healthy periodontitis patients; sodium hypochlorite used adjunctively to SRP in the test group; a control group received the same SRP as the test group either alone or with a placebo; follow-up period ≥ 6 months after initial therapy; report on clinical treatment outcomes, including CAL and/or PD and/or BOP and/or the incidence of adverse events; English language.

At the second stage of selection, all full-text articles identified during the first stage were acquired and evaluated according to the following exclusion criteria: studies including patients with systemic diseases that could influence the outcome of periodontal therapy; studies treating aggressive-periodontitis patients; studies treating periodontitis as a manifestation of systemic diseases; studies not reporting on the clinical treatment outcomes, including changes in CAL and/or PD and/or BOP.

### Data Collection and Data Items

Data extraction templates were used to retrieve general information on the country, study design, periodontal status of included cohorts, follow-up periods, number of patients, patients’ gender, age, smoking status, and tested products (Table 1). The number of patients at baseline and at end of the study, periodontal case definitions, treatment protocols in test and control groups, and clinical outcomes are presented in Table 2. The mean values and standard deviations of changes in PD and BOP reduction and in CAL gain following the treatment in test and control groups were extracted for the data analysis (Table 2).

**Table 1:** Material and methods of the selected studies: country, study design, periodontal status of included cohort, follow-up, sample size, gender, smoking status, age and tested product

Study	Country	Study design	Periodontal status	Follow-up	Number, gender	Smokers	Mean (range) age	Product tested
Bizzarro et al, 2016^1^	The Netherlands	Parallel RCT	Non-treated CP	12 months	56 (36M, 20F)	Included	47.8 ± 9.3	0.5% NaOCl solution
Megally et al, 2020^24^	Switzerland	Parallel RCT	Persistent/recurrent periodontitis	12 months	32 (21M, 11F)	Included	61.9 ± 9.3	Hypochlorite/amino acid gel

CP: chronic periodontitis; F: female; M: male; NaOCl: sodium hypochlorite; NR: not reported; RCT: randomised controlled clinical trial.

**Table 2:** Material and methods of the selected studies: number of participants at baseline and end of the study, periodontal case definition, treatment protocols, changes in PD, CAL and BOP in test and control groups

Study	Participants	Periodontal case	Intervention	PD changes (mm) mean ± SD	CAL changes (mm) mean ± SD	BOP changes (%) mean ± SD	Comments
Bizzarro et al, 2016^1^	**Control** Baseline n = 29; end of the study n = 29 **Test** Baseline n = 27; end of the study n = 27	≥ 2 non-adjacent teeth interproximal attachment loss of ≥ 3 mm; 2 teeth per quadrant with PD ≥ 5mm; > 50%BOP	**Control** SRP+S **Test** SRP+ 0.5% NaOCl	**Control** 1 ± 0.6 **Test** 0.9 ± 0.3 Statistical significance between the groups p = 0.143; compared to baseline p < 0.001	**Control** 0.6 ± 0.5 **Test** 0.5 ± 0.5 Statistical significance between the groups p = 0.243; compared to baseline p < 0.001	**Control** 42.3 ± 16.9 **Test** 41 ± 12.6 Statistical significance between the groups p = 0.635; compared to baseline p < 0.001	NS
Megally et al, 2020^24^	**Contro**l Baseline n = 16; End of the study n = 16 **Test** Baseline n = 16; End of the study n = 16	PD ≥ 5 mm	**Control** Ultrasonic debridement **Test** Ultrasonic debridement + NaOCl/amino acid gel	**Control** 0.85 ± 1.13 **Test** 0.97 ± 1.09 Significance between the groups p = 0.36; compared to baseline p < 0.001	**Control** 0.82 ± 1.33 **Test** 1.02 ± 1.49 Significance between the groups p = 0.31; compared to baseline p < 0.001	-	NS

BOP: bleeding on probing; CAL: clinical attahment level; PD: probing depth; NaOCl: sodium hypochlorite; NS: no significant difference between test and control group.

### Risk of Bias Assessment

The quality of all included studies was assessed during the data extraction process and involved evaluation of the methodological elements that could influence the outcome of each study (Table 3). The Cochrane Collaboration’s 2-part tool for assessing risk of bias was used to assess bias across the studies and identify papers with intrinsic methodological and design flaws.13 The following domains were included: random sequence generation, allocation concealment, blinding of participants and personnel, blinding of outcome assessment, incomplete outcome data, selective reporting. The degree of bias was categorised as low risk if all criteria were met, moderate risk when one criterion was not met, and high risk if two or more criteria were not met.

**Table 3:** Assesment of the risk of bias

Author, year	Random sequence generation	Allocation concealment	Blinding	Incomplete outcome data	Selective reporting	Other bias
Bizzarro et al, 2016^1^	+	+	+	+	+	+
Megally et al, 2020^24^	+	+	?	+	+	+

+ = low risk; ? = unclear risk; - = high risk.

## Results

### Search Results

**Fig 1:**
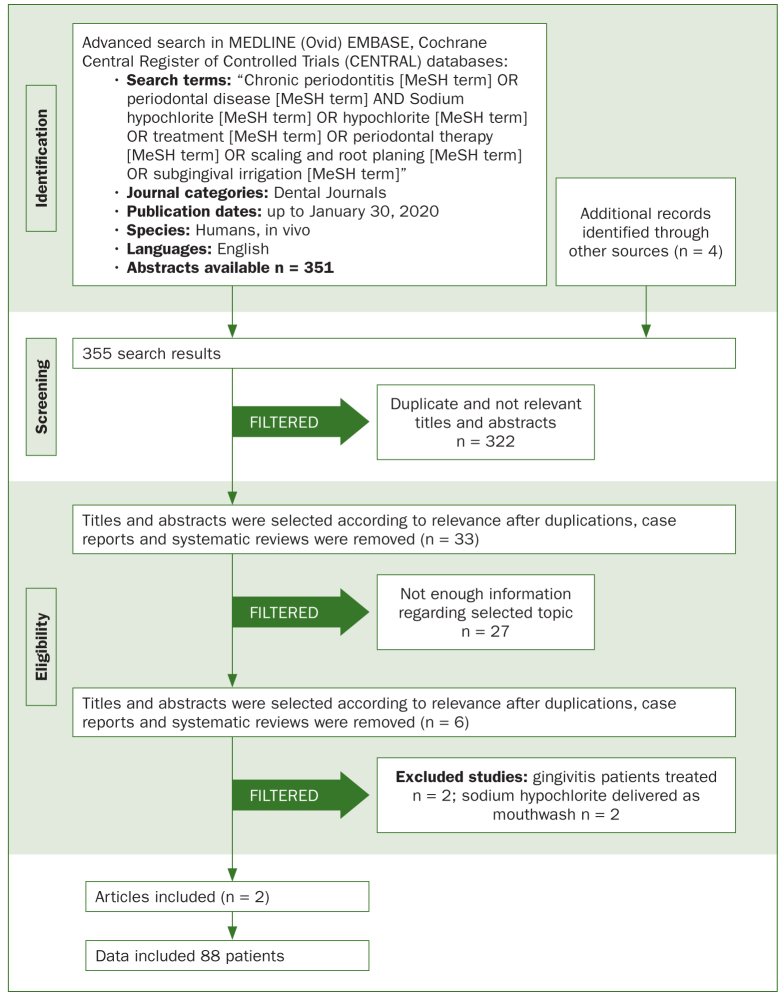
PRISMA flow diagram.

Article review and data extraction were performed according to the PRISMA flow diagram (Figure 1).

The electronic search yielded 351 titles. Four additional articles were identified through the hand search, rendering an initial search selection of 355 records. Following the screening of titles and abstracts, 6 articles were selected for full-text analysis (κ = 0.96). Further, 2 studies treating gingivitis^2,19^ were excluded, and 2 studies where sodium hypochlorite was delivered as a mouthwash^5,7^ were also excluded (κ = 1), resulting in a final selection of 2 articles^1,24^ (κ = 1).

### Quality Assessment

The included studies were classified as low risk of bias for all key domains (Table 3).

### Study Characteristics

#### Study design

Both of the included studies were parallel-arm randomised controlled clinical trials with a follow-up of 12 months. One of the studies included three test groups (i.e. SRP + adjunctive irrigation with 0.5% NaOCl; SRP + 0.5% NaOCl + systemic antibiotics; SRP + adjunctive irrigation with saline + systemic antibiotics). However, due to the adjunctive use of systemic antibiotics following the SRP, only one test group (SRP + adjunctive irrigation with 0.5% NaOCl) could be included in the present analysis.^1^

#### Study population

One of the included studies involved patients with untreated chronic periodontitis,^1^ while the other study included patients diagnosed with recurrent periodontitis who were enrolled in a regular periodontal maintenance program.^24^ In total, 43 patients were treated with adjunctive sodium hypochlorite (test group), and the remaining 56 patients underwent mechanical debridement alone (control).

The mean age of the included patients was 47.95 (9.9) and 61.9 (9.3) years respectively.^1,24^ The ratio of males: females was 36:19 in first of the included studies,^1^ and 21:11 in second.^24^ Both studies were based on patient samples from a European population. Smokers were included in both investigations at proportions ranging from 13%^24^ to 55%.1

Patient-related data are depicted in Table 1.

### Interventions

Treatment protocols in the test and control groups are depicted in Table 2. In both studies, oral hygiene instructions were given to the patients prior to treatment. Additional postoperative antiseptic rinsing was restricted in one study,^24^ whereas patients were prescribed to rinse with 0.12% chlorhexidine in the other.^1^ Subgingival debridement was accomplished by the means of ultrasonic instrumentation and gracey curettes,^1^ or by ultrasonics only.^24^ Sodium hypochlorite was applied as a subgingival 0.5% irrigant in one of the included studies^1^ and as a hypochlorite/amino acid gel in the other.^24^

### Treatment Outcomes

Because only two studies were included, meta-analyses could not be performed.

In both studies, in comparison to the baseline, the investigated clinical parameters statistically significantly improved in both test and control groups. In particular, the mean PD reductions amounted to 1 (±0.6),^1^ and 0.85 (±1.13)^24^ mm in control and 0.9 (±0.30),^1^ and 0.97 (±1.09)^24^ in test groups (p < 0.05). The mean CAL gain was 0.6 (±0.5)^1^ and 0.82 (±1.33)^24^ mm in control and 0.5 (±0.5),^1^ and 1.02 (±1.49)^24^ mm in test groups (p < 0.05). The reduction of BOP scores was reported in one study, with 42.3 (±15)% and 41 (±12.6)% in control and test groups, respectively.^1^ No adverse clinical events were reported for the adjunctively applied sodium hypochlorite.

However, when comparing the test and control treatment approaches, no statistically significant differences in any of the investigated clinical parameters between groups could be detected (p>0.05) (Table2).

## Discussion

The aim of the present systematic review was to assess the existing evidence of the effectiveness of subgingival application of sodium hypochlorite and its clinical benefits in nonsurgical periodontitis treatment. The literature search pointed to limited existing clinical evidence, as only 2 RCTs were eligible for inclusion. Based on our findings, no additional long-term effects in PD reduction, CAL gain or BOP values were detected when sodium hypochlorite was adjunctively used for nonsurgical periodontitis treatment. Additionally, the application of sodium hypochlorite did not cause adverse events.

Sodium hypochlorite was introduced to aid periodontal therapy in the early 1990s.^16^ Today, it is still a primary irrigant in endodontics, but has very limited use in periodontology. It has been reported in in vitro studies that sodium hypochlorite might effectively remove endotoxins from the periodontally involved root surfaces, and act bactericidically not only on periopathogenic bacteria but also on bacterial species associated with peri-implantitis.^2,6,16,18,35^ Moreover, it has been shown to aid in removing the inflamed epithelium from periodontal pockets without detrimental effects on wound healing.^17^ Importantly, it might help establish surfaces favourable for periodontal ligament cell survival, attachment, and spreading.^36^

The clinical benefits of sodium hypochlorite have been shown in previous clinical studies where it was employed as a self-care oral rinse. Specifically, it was demonstrated that as an oral mouthwash, sodium hypochlorite helped reduce gingivitis and bleeding of periodontal pockets.^2,7,19^ Moreover, it inhibited plaque accumulation and stabilised the plaque pH.^2,5,19^ On the basis of these studies, it can be assumed that sodium hypochlorite might constitute a valuable antiseptic in periodontal self-care.

In the present review, sodium hypochlorite was employed as a subgingival antiseptic in two different formulations – as a subgingival irrigant^1^ and as a sodium hypochlorite/amino acid gel.^24^ The importance of a delivery system of an antiseptic has been highlighted in previous systematic reviews.^9,22^ In particular, Hanes and Purvis^9^ emphasised that sustained-released systems, not irrigated, should be employed to maintain therapeutic concentrations of antimicrobials at the local site. Accordingly, the authors of one of the included studies^1^ indicated that a short-lived antimicrobial effect of NaOCl solution disappeared in the long term, and possibly led to bacterial recolonisation of the pockets.

Nevertheless, the delivery form of sodium hypochlorite in a gel formulation also failed to show significant clinical benefits compared to ultrasonic instrumentation alone.^24^ Nevertheless, it should be noted that greater PD reduction in initially deep residual pockets (≥7 mm) was observed in the adjunctive sodium hypochlorite/amino acid gel group. In particular, following treatment, only one residual pocket of ≥ 7 mm was still detectable in a test group, whereas six compromised sites persisted in a control group. Based on this observation, aminoacid/sodiumhypochlorite gel might be an effective adjunctive material in initially deep, persistent periodontal pockets.

## Conclusion

The available data have failed to show any additional clinical benefit following the use of sodium hypochlorite in conjunction with nonsurgical periodontal therapy.
